# Current and future applications of brain magnetic resonance imaging in ARSACS

**DOI:** 10.1007/s12311-025-01842-x

**Published:** 2025-04-30

**Authors:** Alessandra Scaravilli, Davide Negroni, Claudio Senatore, Filippo Maria Santorelli, Sirio Cocozza

**Affiliations:** 1 Department of Advanced Biomedical Sciences, University of Naples “Federico II”, Via Pansini 5, Naples, 80131 Italy; 2 Department of Molecular Medicine, IRCCS Stella Maris Foundation, Pisa, Italy

**Keywords:** ARSACS, Ataxia, Magnetic resonance imaging, Brain

## Abstract

Magnetic Resonance Imaging (MRI) is a tool with an unquestionable role in the study of neurodegenerative disorders, both for diagnostic purposes and for its ability of providing imaging-derived biomarkers with a growing central role as reliable outcomes in clinical trials. This is even more relevant when dealing with rare disorders such as the Autosomal Recessive Spastic Ataxia of Charlevoix-Saguenay (ARSACS), in which the search of diagnostic and prognostic biomarker is crucial. Due to the rarity of this condition, a comprehensive knowledge of MRI signs observed in ARSACS is lacking. Furthermore, many domains remain still unexplored in ARSACS, especially with reference to the application of advanced imaging techniques that could shed light on the pathophysiological mechanisms of brain damage in this disorder. In this review, after a brief introduction on the major conventional and advanced MRI techniques that can used for diagnostic and research purposes, we present current neuroradiological knowledge in ARSACS. Having discussed strength and weak points of conventional and advanced imaging findings, we also suggest possible future research in this neurologically complex clinical condition.

## Introduction

Autosomal Recessive Spastic Ataxia of Charlevoix-Saguenay (ARSACS) is a rare genetic disorder caused by mutations in the *SACS* gene [1]. Initially described in a cluster of families in the Charlevoix-Saguenay-Lac-Saint-Jean region of Quebec [2], where a common mutation reaches an incidence at birth estimated at 1 in 1,932 [3], ARSACS has now been described worldwide and it is considered the second more common form of inherited ataxia after Friedreich ataxia [4]. ARSACS is associated with loss-of-function variants in the *SACS* gene [5], leading to depletion of *sacsin*, a ∼520 kDa large multidomain protein widely expressed in layer 5 corticospinal neurons, anterior commissure, anterodorsal nuclei of thalami, Purkinje cells and brainstem nuclei [6, 7].

Clinically, ARSACS is a slowly progressive disease with a relatively heterogeneous clinical features and subtle phenotypic variability between Quebecois and non-Quebec patients [8]. The age of onset typically occurs between 12 and 18 months of age, although late infantile, juvenile and early adult onset have also been described [9, 10]. The classic phenotype present a triad of manifestations consisting in a slowly progressive cerebellar ataxia, lower limb spasticity and peripheral neuropathy, although the absence of one of these features do not contradict the diagnosis [9–11]. Furthermore, features as hearing loss, intellectual disability and myoclonic epilepsy have been observed in addition to these classic symptoms [12–14]. In Quebecois, hypermyelination of retinal fibres on ophthalmologic examination and optical coherence tomography (OCT) scans represents a constant feature, but it may be absent in non-Quebec patients [15].

Although the clinical suspect of ARSACS is corroborated by genetic testing and the final diagnosis relies upon identification of biallelic loss-of-function variants in *SACS* [4], Magnetic Resonance Imaging (MRI) of the brain represent a crucial step in the diagnostic flowchart. Indeed, the identification by neuroradiologist of typical features can significantly shorten the diagnostic process by prioritizing genetic tests [16]. MRI is the only technique able to capture and reliably evaluate in-vivo information of cerebral macro- and microstructures or the neurochemical profile as disease progresses via the application of several advanced techniques [17–19] and it represent a potential tool for imaging-derived biomarkers to serve as outcomes to the clinical trials that will emerge in the near future [20].

Given this background, aim of this work is to review current knowledge on brain MRI findings in ARSACS. We first introduce the main sequences currently used for diagnostic purposes (and how these should be applied in clinical practice), and the major advanced imaging techniques with a likelihood to investigate the pathophysiology of neurodegeneration in ARSACS. Afterwards, the most relevant conventional and advanced imaging features of ARSACS are presented, with the final aim of providing useful insight for neuroradiologists and physicians and stimulate future research increasing our knowledge of the pathophysiology underlying this rare disease.

## Conventional and advanced MRI sequences for the study of ARSACS

The first step for an accurate MRI evaluation of a patient presenting with clinical symptoms suggestive of an inherited ataxia is the acquisition of a proper protocol. Acquisitions should be performed using scanners of at least 1.5 Tesla (T). Scanners of 3T magnetic field intensity are preferable, given that the high field strength allows for an increased sensitivity able to capture small alterations due to the higher spatial resolution [20]. A proper MR protocol should include a 3D-T1-weighted sequence with a voxel resolution of at least 1 mm isotropic, that allows reconstruction of images on the three different plans via Multiplanar Reconstruction (MPR) analysis, to thoroughly evaluate the entire morphology of the brain and atrophy. If this sequence is not available on the scanner, a Spin Echo (SE) T1-weighted acquired on the sagittal plane is suggested, preferably with a slice thickness not superior to 3 mm, to allow for an accurate evaluation of the anatomy of pons and cerebellar vermis. Along with T1-weighted sequences, to detect the peculiar signal abnormalities described in these patients, a MR protocol in ARSACS must include T2-weighted sequences and Fluid Attenuated Inversion Recovery (FLAIR) sequence, being the most sensitive sequence for the detection of brain signal changes. It is noteworthy to mention that one of the major limitations of FLAIR imaging is represented by the presence of artefacts from the inflow of non-inverted cerebrospinal fluid and from pulsatile motion [21, 22], which might be even more evident in the infratentorial compartment, producing false-positive or false-negative interpretation of signal changes in FLAIR images [23]. Volumetric 3D-FLAIR sequences, that are virtually unimpaired by these artifacts [24], leveraging other advantages such as MPR and volume measurements [25] and are therefore suggested in the MR protocol of ARSACS patients compared to their 2D counterparts. Finally, Diffusion (DWI) and Susceptibility Weighted Imaging (SWI) sequences are also mandatory, to detect possible areas of cytotoxic edema and microbleeds respectively, both infrequent findings in ARSACS but crucial information in the overall neuroradiological diagnostic workup of patients presenting with ataxia symptoms.

In a research setting, several advanced MRI sequences can be used, to investigate the different aspects of the pathophysiological changes occurring in ARSACS. In particular, diffusion MRI (dMRI), based on the anisotropic diffusion of water molecules in tissues and therefore, allow for the evaluation of the integrity of white matter (WM) bundles [26, 27]. Different biophysical models can be applied to dMRI to extract information on WM microstructure, with Diffusion Tensor Imaging (DTI) that represents the most widely used model [28]. From the application of this model several parameters can be extracted, including the fractional anisotropy (FA), a scalar value that indicates how much the diffusion of water in a tissue deviates from the expected isotropic condition, the mean diffusivity (MD), inversely correlated to FA and often used as a proxy of the presence of oedema, as well as radial (RD) and axial (AD) diffusivities used as indices of demyelination and axonal damage, respectively [29]. Along with brain macro- and microstructure, its function can also be investigated in-vivo via the application of advanced MRI sequences such as functional MRI (fMRI), that employs subtle changes in local blood oxygenation levels induced by neuronal activation following a motor or cognitive task, or due to their spontaneous fluctuations signal over time without the need of an active task [30]. Finally, the MR spectroscopy (MRS) is the only technique able to non-invasively quantify endogenous neurochemical profiles of neurodegeneration, such as membrane metabolism, oxidative stress, neuronal integrity, glial activation, and energy deficits [31].

Recent guidelines to facilitate and harmonize advanced MRI data acquisition for clinical research and trial readiness in inherited ataxias have been recapitulated by the MRI Biomarkers Working Group of the Ataxia Global Initiative [20].

## Conventional MRI imaging in ARSACS

Current knowledge about the typical conventional MRI features of ARSACS is derived from single-case studies or reports of small series of patients, a common theme in rare diseases. Among the abovementioned studies, there are few imaging features common to all ARSACS patients, such as the occurrence of cerebellar atrophy, with a more prominent, significant and in some cases selective involvement of the superior vermis [9, 32–42]. Atrophy of this cerebellar area, best seen on coronal or midline sagittal images, results from a neuroradiological perspective in a widening of the cerebellar fissures with an increased representation of the cerebrospinal fluid spaces following the loss of normal cerebellar architecture. Nonetheless, it is noteworthy to mention that atrophy of the cerebellar hemispheres has also been described and can be observed in these patients [36, 38, 43–45]. Along with this relatively selective area of atrophy, another volumetric hallmark of ARSACS is the “bulky” appearance of the pons, increased in volume and coupled to a thickening of proximal portion of the middle cerebellar peduncles (MCP) [9, 33, 34, 36, 37, 39, 40, 42]. The combination of these two neuroradiological signs, and in particular their ratio, has been recently described as the Magnetic Resonance Index for the Assessment and Recognition of patients harboring *SACS* mutations (MRI-ARSACS), a novel diagnostic tool for the early identification of patients undergoing an MRI scan [46]. Additional volumetric changes affecting the infratentorial compartment reported in some literature cases are the thinning of the superior cerebellar peduncles (SCP) [33] and the medulla oblongata [33, 40], along with a reduced antero-posterior diameter of the cervical [34, 38, 40, 47–49] or dorsal [37, 50] spinal cord. Finally, a relatively frequent association with the presence of a posterior fossa arachnoid cysts has also been described [9, 39, 51, 52].

Along with volume changes, also signal modifications of the infratentorial compartment have been reported in ARSACS, and similarly to the volumetric counterpart mainly affecting the pons. Indeed, the evidence of parallel T2-weighted or FLAIR hypointense stripes in the pons on either side of the midline, better appreciated in the axial planes, has been frequently described in a vast majority of ARSACS patients and undergo the name of “pontine stripes” [9, 32–38, 40, 42]. These stripes are in some cases coupled to T2-weighted or FLAIR hyperintensity of the lateral part of the pons [9, 33, 39, 40, 42, 47]. Although these have to be considered, to date, a typical MRI finding of ARSACS, it is noteworthy to highlight they are not described in all patients in the literature [41, 53–56]. Furthermore, there is no real agreement on which is the best MRI sequence for an adequate assessment of this signal alteration of the pons, being documented by some authors only in FLAIR [9] or T2-weighted images [35–37, 42], and rarely in both sequences [32–34, 38, 40]. For this reason, future studies should aim to define which sequence should be used in clinical routine practice, the real incidence of these signal changes, and their diagnostic accuracy compared to the other signs already present in literature [46]. Along with these pontine stripes (and the associated T2-weighted hyperintensity of the lateral portion of the pons), a mild T2-weighted hyperintensity of dentate nuclei [33, 37] has been anecdotally reported as an additional signal change affecting the infratentorial compartment.

The degree of involvement extends beyond the infratentorial areas, with the occurrence of alterations of the supratentorial compartment in ARSACS patients detectable with conventional MRI. These include the occurrence of a diffuse cortical atrophy [57], with an apparent more selective bilateral parietal cerebral atrophy described in many reports [9, 47, 52]. Furthermore, volumetric changes in the supratentorial WM have also been reported, with a thinning of the corpus callosum [33, 57], especially in its posterior mid-body component [9, 38, 39, 47, 52]. Volumetric changes in the corpus callosum have also been recently reported in a murine model of the disease, showing a selective early involvement of the genu and a significant thinning of the splenium in later stages of disease [58].

Finally, signal changes of the supratentorial compartment are less common than their infratentorial counterparts, mostly characterized by a well-defined thin T2-weighted hyperintense rim along the lateral margin of the thalami, that can be described as “thalamic rim sign” [33, 38, 39], and hypothesized to be related to degeneration of fibres of the external thalamic lamina and reticular thalamic nucleus.

**Fig. 1:**
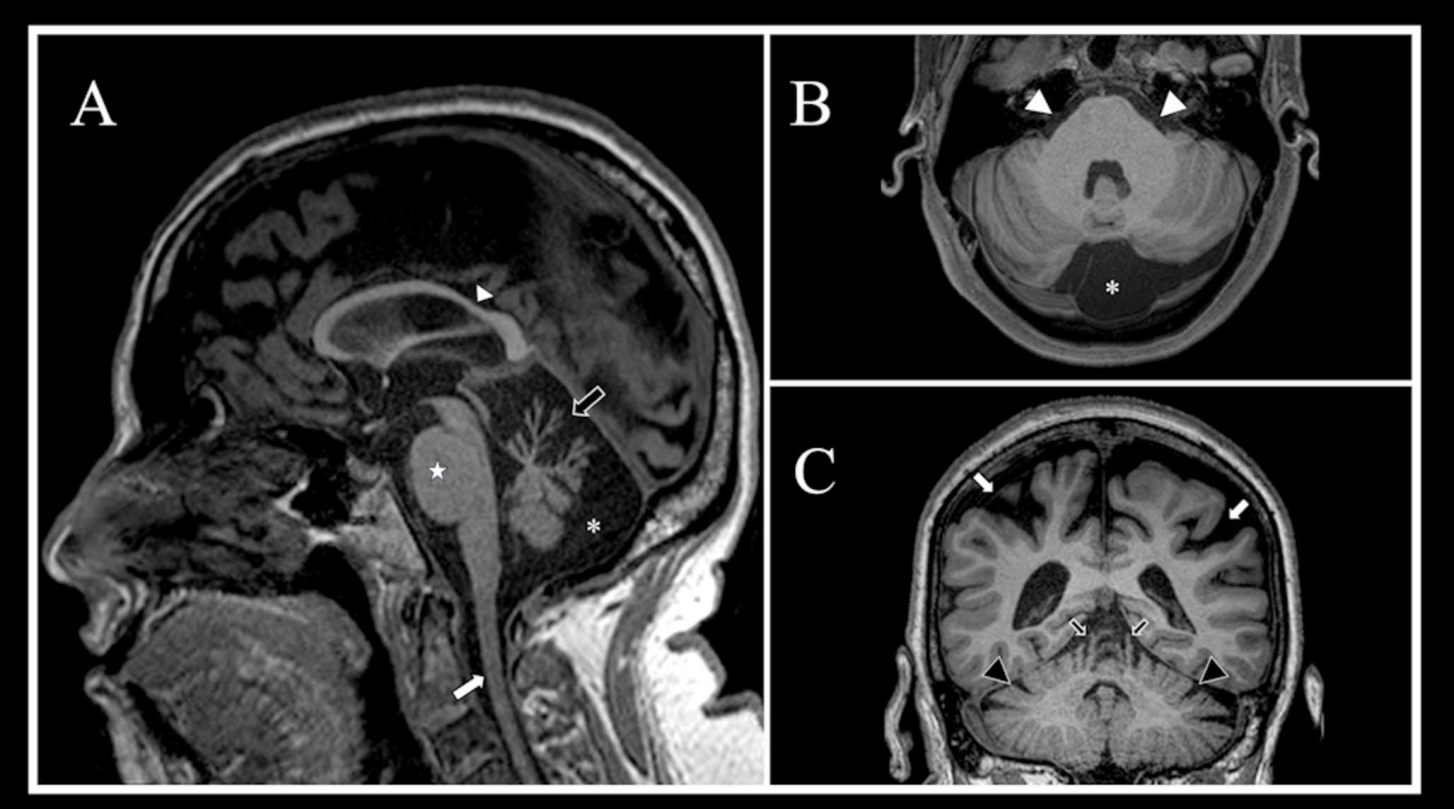
Patterns of atrophy detectable via conventional MRI in ARSACS. A sagittal midline T1-weighted image (**A**) showing in an ARSACS patient the typical occurrence of superior vermis atrophy (*black arrow*) along with a “bulky” appearance of the pons (*white star*). Other imaging findings include a thinning of the mid-posterior segment of corpus callosum (*white arrowhead*), a mild atrophy of the upper cervical cord (*white arrow*) and the evidence of a posterior fossa arachnoid cyst (*white asterisk*), with the latter finding also visible in the axial multiplanar reconstruction in (**B**), where is also possible to appreciate the thickening of middle cerebellar peduncles (*white arrowheads*). Finally, a coronal multiplanar reconstruction (**C**) confirming the occurrence of a significant superior vermis atrophy (*black arrow*s), coupled to a mild degree of cerebellar hemispheres (*black arrowheads*) and, more pronounced, biparietal (*white arrows*) atrophy

**Fig. 2:**
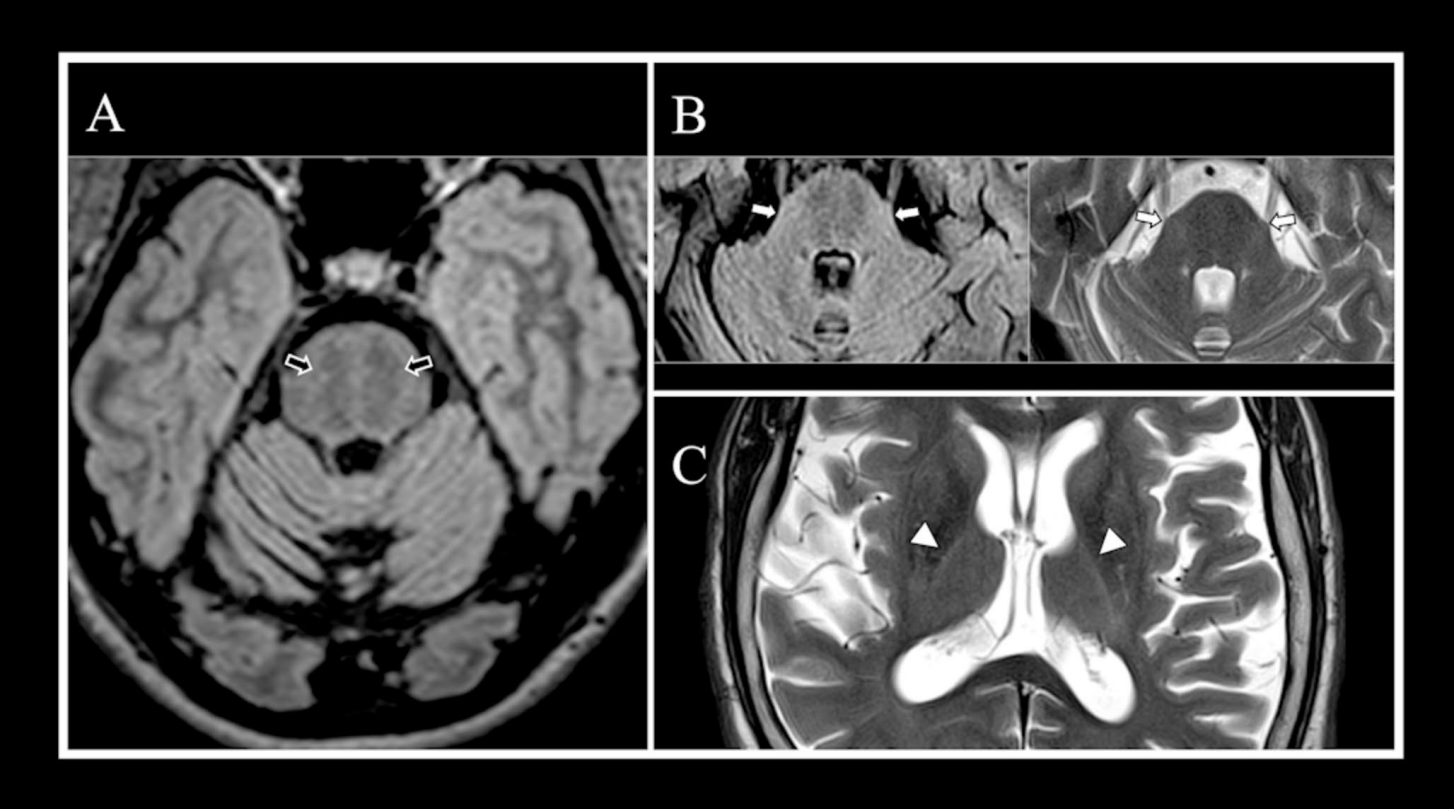
Patterns of signal changes detectable via conventional MRI in ARSACS. Axial multiplanar reconstruction (**A**) of a 3D Fluid Attenuated Inversion Recovery (FLAIR) sequence showing in an ARSACS patient the occurrence of the “pontine stripes” (*black arrows*). Axial (**B**) image of 2D FLAIR (*left panel*) showing a mild hyperintensity in the lateral portion of the pons merging in middle cerebellar peduncles, confirmed evaluating the turbo spin echo T2-weighted sequence (*right panel*). On both images, is possible to observe the thickening of middle cerebellar peduncles (*white arrows*). An axial T2-weighted image (**C**) shows the presence of a bilateral hyperintensity of lateral thalami, described as “thalamic rim sign” (*white arrowheads*)

Examples of the main volumetric and signal changes reported in ARSACS are shown in Figs. 1 and 2, respectively, while a summary of key conventional imaging findings in this condition is shown in Table 1.

**Table 1:** Conventional MRI findings reported in ARSACS

		Volume changes	Signal changes	Other significant MRI changes
*Infratentorial*		Cerebellar atrophy, with a more prominent, significant and in some cases selective involvement of the superior vermis “Bulky” appearance of the pons Thickening of proximal portion of the MCP Thinning of SCP and medulla oblongata less frequently reported	“Pontine stipes”, described as parallel T2-weighted or FLAIR hypointense stripes in the pons on either side of the midline T2-weighted or FLAIR hyperintensity of the lateral part of the pons T2-weighted hyperintensity of DN anecdotally reported	Reported high association with posterior fossa arachnoid cysts
*Supratentorial*		Some degree of cortical atrophy, with a more selective bilateral parietal involvement Thinning of corpus callosum, with a prominent involvement of the posterior mid-body portion	T2-weighted hyperintense rim along the lateral margin of the thalami	No additional significant associations reported to date
*Spine*		Cervical and dorsal spinal cord atrophy might be observed	No signal changes reported to date	No additional significant associations reported to date

DN = dentate nuclei; MPC = middle cerebellar peduncles; SCP = superior cerebellar peduncles

## Advanced MRI imaging in ARSACS

Application of advanced neuroimaging techniques in ARSACS is a field almost completely unexplored. Indeed, only few studies are currently present in the literature, mainly dealing with DTI and tractography applications in single subjects [57, 59, 60] or in relatively small groups of patients [33, 36, 49, 61, 62]. Based on the knowledge arising from the major findings observed on conventional imaging, these studies have been mainly focused on the alterations of the corticospinal tract (CST) and the infratentorial compartment, and in particular of the pons, with the aim of shedding light on the pathophysiological significance of these observed changes.

From a macrostructural perspective, the major DTI finding described is represented by the prominent occurrence, at the level of the pontine basis and tegmentum, of latero-laterally oriented fibers, which have been interpreted by some authors as hypertrophic transverse pontine fibers [33, 36, 42, 57, 60] or, more probably, as pontocerebellar fibers [49, 61, 62]. This finding has been also observed in combination with a thin and abnormally placed portion of the CST at the pons [42, 57], as well as an abnormally thick MCP [33, 49, 59–62]. These findings were confirmed also when tractography images were reconstructed from diffusion data, showing a relative thinning of the CST in the midbrain and medulla oblongata, also coupled to a possibly artefactual interruption of the CST at the pons [36, 62]. These features also combine with a normal size and location of the pyramids at the medulla, and a large number of pontocerebellar fibers at the basis and tegmentum of the pons and MCP compressing the CST [33, 49, 61].

From a microstructural perspective, prominent latero-laterally oriented fibers detected at the level of the pons revealed higher FA [33], lower RD and higher AD values [36] in ARSACS compared to healthy controls, with these changes likely implying axonal hypertrophy (or possibly hypermyelination phenomena) of the specific fibers. Furthermore, the CST portion in the brainstem seemed to reveal a significantly lower FA values, coupled with an increase in RD and MD in the same individuals [36], whereas the portion of CST cranial to the midbrain level showed a reduced FA bilaterally (and an increase in MD only for the right CST) [33]. As the lack of recognizable decussation of the SCP has been reported only in one study, coupled to a marked FA reduction and increased MD in SCP [33], this finding need to be further corroborated as specific of ARSACS. Furthermore, microstructural alterations in pontocerebellar fibers, connecting pontine nuclei to the granular layer of the cerebellar cortex, have been hypothesized as possibly leading to an excessive amount of glutamate determining excitotoxic phenomena within the cerebellar cortex, thus resulting in neuronal death [49, 61] and ultimately WM loss, as demonstrated by alteration in FA and RD in cerebellar WM and vermis [36]. Together, these phenomena may account, at least in part, for the early onset of spasticity and progressive ataxia observed in ARSACS patients.

Macro- and microstructural changes detectable via dMRI have been interpreted as the counterpart of the signal changes detailed in conventional imaging, and put forward a possible neurodevelopmental hypothesis in ARSACS [61]. Indeed, it has been hypothesized an early role of *sacsin* in guiding neurogenesis and a later progressive impact on the process of neurodegeneration. Accordingly, it has been proposed that the increase representation of pontocerebellar fibers, potentially present since the embryonic period, and the consequent mechanical compression of CST bundles might explain early-onset spasticity found in most ARSACS patients [33]. If this hypothesis were correct, we might expect an almost invariable early representation of abnormal signal intensity while performing natural history studies (NHS), and a possible secondary degeneration of CST (as currently only suggested by the microstructural damage of CST bundles observed right above and below the pons). A multicenter multimodal longitudinal NHS will help to offer proper answers [63]. To date, only few studies have explored possible alterations of the WM microstructure of the supratentorial compartment [33, 36, 64], reporting reductions in FA and increased MD in different major brain fiber bundles, such as the forceps minor and major, the superior longitudinal fasciculum and the cingulum [33]. Furthermore, extensive reductions in FA, accompanied by increased RD and reduced AD were also reported, via tract-based-spatial statistics analysis, affecting the entire corpus callosum and other major WM tracts such as the inferior fronto-occipital fasciculi, the corona radiata or internal capsules [36]. These findings suggest that widespread WM changes might occur in ARSACS, mainly sustained by demyelination, which extends beyond motor pathways and involve key associative WM bundles. These findings were recently confirmed in a study involving a large cohort of ARSACS patients from the internation collaborative research project PROSPAX [63] which showed severe and widespread WM involvement, both at a macrostructural and microstructural level, with a more pronounced involvement of the commissural fibers [64].

Finally, very scattered evidences are available in literature about possible neurochemical changes in ARSACS [49, 65]. Indeed, to date only two studies have investigated possible neurochemical changes in this condition, both failing to find significant alteration in the infratentorial compartment [49, 65], while an apparent increase in myoinositol concentration at the level at the frontal lobe has been reported in a small group of four ARSACS patients [65].

On the other hand, no fMRI studies have ever been performed in ARSACS, leaving unanswered questions about the possible functional counterparts of the observed macro- and microstructural changes, phenomena often occurring in other inherited ataxias [66–72].

**Fig. 3:**
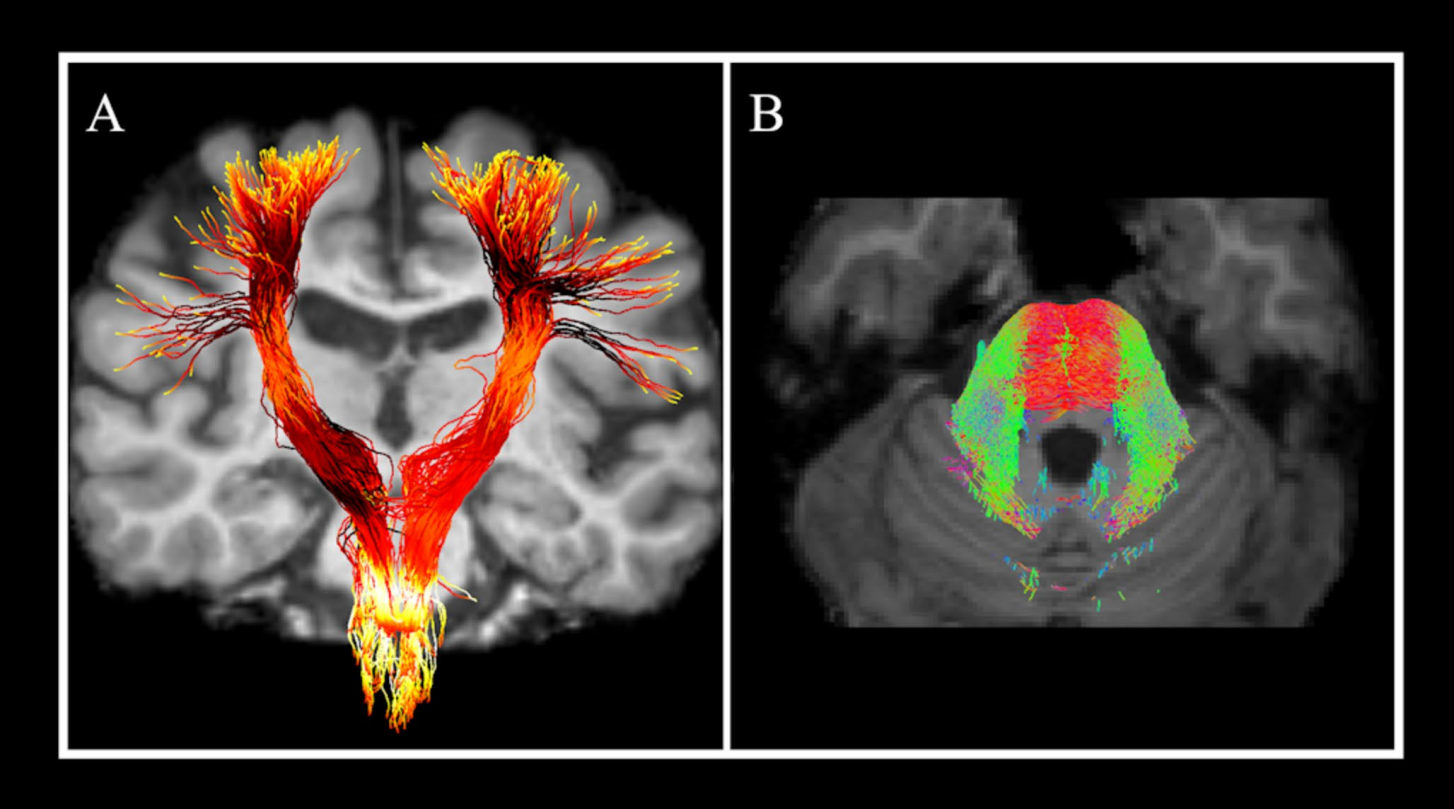
An example of advanced imaging findings in ARSACS. Tractography reconstruction from a diffusion MRI sequence using a probabilistic approach of the corticospinal tract (**A**) and of the bundles at the level of the pontine region (**B**), with the latter image showing an over-representation of the latero-laterally oriented fibres that fill nearly the whole pons (*red*), along with thickened middle cerebellar peduncles (*green*) in an ARSACS patient

An example of the application of advanced MRI technique for the study of ARSACS is shown in Fig. 3, while a summary of advanced imaging findings in these patients is shown in Table 2.

**Table 2:** Advanced MRI findings reported in ARSACS

	Infratentorial		Supratentorial
*dMRI*	*Macrostructure*	*Microstructure*	Widespread WM changes, mainly sustained by demyelination, extending beyond motor pathways and involving key associative bundles Decreased FA and AD, and increased RD, in the entire corpus callosum, the corona radiata and internal capsules Decreased FA and increased MD in forceps minor and major, superior longitudinal fasciculum and cingulum
	Prominent latero-laterally oriented fibers at the level of the pontine basis and tegmentum Abnormally thickened MCP Thinned CST at the level of the pons, compressed by pontocerebellar fibers	Increased FA and AD, and decreased RD, in latero-laterally oriented pontine fibers Decreased FA, with increased RD and MD, in the brainstem portion of the CST Decreased FA of the CST cranial to the midbrain Changes in FA and RD values at the level of cerebellar WM and vermis	
*MRS*	Two studies using single-voxel MRS failed to prove significant neurochemical alterations of the pons		Only one study using single-voxel MRS showed an increase in [mI] in the left frontal lobe

AD = axial diffusivity; CST = corticospinal tract; dMRI = diffusion MRI; FA = fractional anisotropy; fMRI = functional MRI; MCP = middle cerebellar peduncles; MD = mean diffusivity; MRS = MR spectroscopy; RD = radial diffusivity; WM = white matter; [mI] = myoinositol concentration

## Conclusion

We provided a comprehensive review of the conventional and advanced MRI findings in ARSACS. Although this is a rare disorder, a clear knowledge of neuroimaging findings is crucial in this condition, given that some highly suggestive (i.e., superior vermis cerebellar atrophy), almost pathognomonic (i.e., “bulky” appearance of the pons), MRI findings have been described in this condition and thus might be very useful at the time of diagnosis. On the other hand, the application of advanced imaging techniques has only partly expanded our knowledge about the physiopathology of this condition, strengthening a neurodevelopmental hypothesis of ARSACS coupled to signs of neurodegeneration reflected by significant and widespread WM damage, mainly sustained by demyelination. However, different domains still remain widely unexplored in ARSACS, with the application of other advanced imaging techniques that should fill the gap in knowledge in the future years by integrating multimodal imaging techniques. At the very end, this collective knowledge may inform clinical management planning, look over progression with the ambition to halt or delay neuronal death, and support new outcomes in the development of targeted engagement interventions.

## Data Availability

No datasets were generated or analysed during the current study.

## Abbreviations

ARSACS: Autosomal recessive spastic ataxia of Charlevoix-Saguenay
CST: Corticospinal tract
DTI: Diffusion Tensor Imaging
FLAIR: Fluid Attenuated Inversion Recovery
MCP: Middle Cerebellar Peduncles
MRI: Magnetic Resonance Imaging
SCP: Superior Cerebellar Peduncles
WM: White Matter
